# Madame La Chapelle

**Published:** 1822-02

**Authors:** 


### MADAME LA CHAPELLE.

The Journal of Foreign Medicine, published in this country, introduced, a few months since, Madame la Chapelle to the notice of the medical profession in England, not only as a most eminent and experienced midwife, but as the accomplished and highly-gifted author of a practical work on Obstetricism. Our predecessors, in conducting the London Medical and Physical Journal, have also, on many occasions, mentioned this lady's name, while recording the many improvements that have taken place, in France, in the department of obstetrical tuition for female pupils. It is with regret, therefore, that we discharge the painful duty of announcing her death. This event took place on the 4th of October, at the Hospital of la Maternité, of which she had been the *sage femme en chef* for a great number of years, and teacher of practical midwifery. Her mortal remains were followed to the grave by upwards of two hundred female pupils, and a vast concourse of members of the faculty. Mademoiselle Holleville, her principal assistant and friend, pronounced a suitable eulogium at her tomb, in which she briefly recapitulated the virtues and the amiable qualities of her departed mistress. Madame la Chapelle has published two works, of which public report speaks highly: 1, Pratique des Accouchemens; 2, Recherches sur les Maladies des nouveaux-nés. She possessed great tact, unerring judgment, much feeling, and a most charitable disposition towards the suffering objects of her care. She had acquired great dexterity in the use of obstetrical instruments, and enjoyed a well-merited reputation as a private practitioner. We, who had the pleasure of knowing her worth during our attendance, of nearly two years, at the vast hospital, the chief management of which was entrusted to her care, readily join in the chorus of her afflicted and eulogizing friends.

---

